# The Swimmer's view: does it really show what it is supposed to show? A retrospective study

**DOI:** 10.1186/1471-2342-8-2

**Published:** 2008-01-15

**Authors:** Ulfin Rethnam, Rajam SU Yesupalan, Salah S Bastawrous

**Affiliations:** 1 Department of Orthopaedics, Glan Clwyd Hospital, Bodelwyddan, UK

## Abstract

**Background:**

One of the basic principles in the primary survey of a trauma patient is immobilisation of the cervical spine till cleared of any injury. Lateral cervical spine radiograph is one of the important initial radiographic assessments. More than often additional radiographs like the Swimmer's view are necessary for adequate visualisation of the cervical spine. How good is the Swimmer's view in visualisation of the cervical spine after an inadequate lateral cervical spine radiograph?

**Methods:**

100 Swimmer's view radiographs randomly selected over a 2 year period in trauma patients were included for the study. All the patients had inadequate lateral cervical spine radiographs. The radiographs were assessed with regards to their adequacy by a single observer. The criteria for adequacy were adequate visualisation of the C7 body, C7/T1 junction and the soft tissue shadow.

**Results:**

Only 55% of the radiographs were adequate. None of the inadequate radiographs provided adequate visualisation of the C7 body and the C7/T1 junction. In 42.2% radiographs the soft tissue shadow was unclear. Poor exposure accounted for 53% of the inadequacies while overlapping bones accounted for the rest.

**Conclusion:**

Clearing the cervical spine prior to removing triple immobilisation is essential in a trauma patient. This needs adequate visualisation from C1 to C7/T1 junction. In our study Swimmer's views did not satisfactorily provide adequate visualisation of the cervical spine in trauma patients. We recommend screening the cervical spine by a CT scan when the cervical spine lateral radiographs and Swimmer's views are inadequate.

## Background

Lateral cervical spine radiograph is one of the important initial radiographic assessments among the three view series in the trauma patient. An adequate lateral cervical spine radiograph is a valuable projection in detecting cervical spine injuries. The importance of visualizing the C7-T1 junction in a patient with suspected cervical spine injury cannot be understated. Visualising the cervical spine from C1 to C7/T1 junction is of utmost importance to avoid neurological deficit due to missed cervical spine injuries. Missing a subluxation or dislocation at this junction can have dire consequences for the patient. Traditionally the Swimmer's view is used for visualizing the C7-T1 junction. It is used as an adjunct to lateral cervical spine radiographs.

The Swimmer's view is the preferred additional view when the lateral cervical spine radiograph is inadequate (the C7-T1 junction is not clearly visualised). In trauma situations getting an adequate lateral cervical spine is a difficult proposition especially when the cervical spine is triply immobilized. Thus the use of Swimmer's view has increased. Does the Swimmer's view adequately reveal the C7-T1 junction? The aim of our study was to assess this.

## Methods

This was a retrospective study conducted in a district hospital. Over a two year period 100 Swimmer's views from the radiology archiving system were selected for the study. Swimmer's views taken following inadequate lateral cervical spine radiographs in trauma patients were included in the study. Radiographs taken in non trauma patients were excluded from the study. The radiographs were assessed on the digital imaging software Synapse. The selected Swimmer's views were assessed for adequacy. The criteria for adequacy were:

▪ Visualization of the C7 – T1 junction

▪ Visualization of the C7 & T1 vertebral body

▪ Visualization of the soft tissues anterior to the C7 & T1 vertebral bodies.

Data was collected from the archiving system (Synapse). This software allowed better visualization of radiographs by allowing change to the image quality for assessment of the soft tissues, bones and by adjusting the contrast of the image. The radiographs were assessed according to the criteria of adequacy mentioned above. Radiographs were deemed inadequate if there was improper visualization of any of the three structures: the C7 – T1 junction, the C7 & T1 vertebral body and the soft tissues anterior to the C7 & T1 vertebral bodies. Image settings were adjusted using the software (Synapse) for better visualization. After assessment of adequacy, the reasons for inadequacy were documented along with a count up of the inadequate radiographs among the Swimmer's views.

## Results

**Figure 1:**
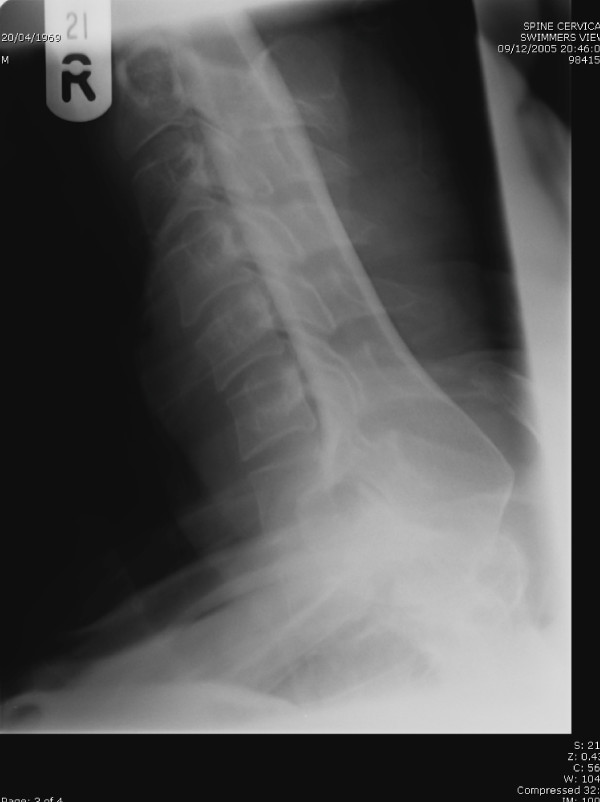
Excellent Swimmer's view. Adequate visualisation of C7T1 junction, C7 & T1 bodies, soft tissues.

**Figure 2:**
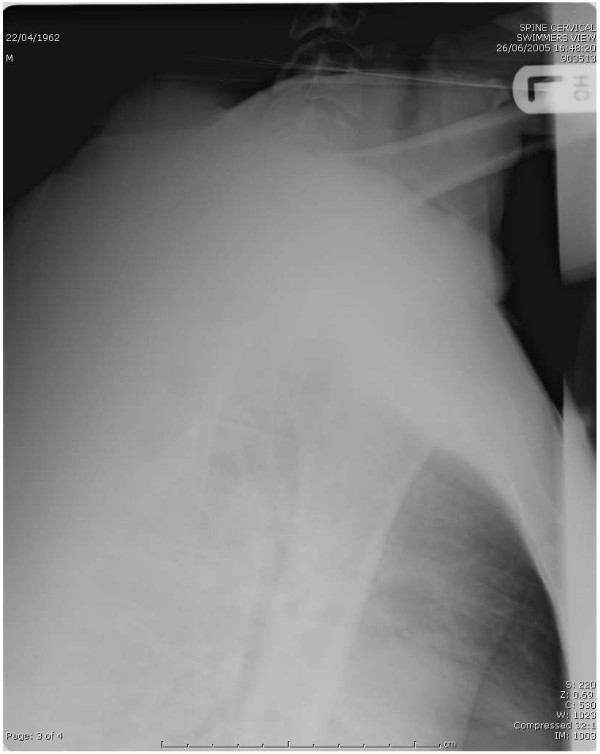
Inadequate swimmer's view. C7 and T1 bodies not visualised. C7/T1junction not seen. Soft tissues not clear. Poor exposure.

100 Swimmer's views were included in the study. 62 patients had concomitant injuries (femoral, tibial, ankle and upper limb fractures) while the remaining patients were suspected to have cervical spine injuries. 55/100 (55%) radiographs were found to be adequate (Figure 1). 45/100 (45%) radiographs were classified as inadequate (Figure 2). Among the inadequate radiographs, the C7-T1 junction and the bodies of C7 and T1 vertebrae were not clearly visualized in all radiographs and the soft tissues were not clear in 19/45 (42.2%) radiographs. The reason for inadequacy were poor exposure in 24/45 (53.3%) radiographs and overlapping bone (humerus & clavicle) in 21/45 (46.6%) radiographs (Table 1). No radiologically significant cervical spine injuries were detected from any of the radiographs assessed or CT scans done following inadequate plain radiographs.

**Table 1:** Swimmer's radiographs – inadequacies and reasons for inadequacy

Swimmer's view n = 100	Adequate – 55/100 (55%)	Inadequate – 45/100 (45%)
Inadequate Swimmer's n = 45	C7/T1 junction & body not clear – 45/45 (100%)	Soft tissues not clear – 19/45 (42.2%)
Reason for inadequacy	Poor exposure 24/45 (53.3%)	Overlapping bones – 21/45 (46.6%)

## Discussion

One of the basic principles in the primary survey of a trauma patient is immobilisation of the cervical spine till cleared of any injury. The lateral cervical spine radiograph is part of the initial radiological survey for trauma patients according to the Advanced Trauma Life Support (ATLS) teachings. The lateral cervical spine radiograph is one of the important initial radiographic assessments for the cervical spine in trauma. Studies have mentioned varied negative predictive values of three view cervical spine series (cervical spine anteroposterior, lateral & odontoid peg view) in trauma patients (93% – 98%) although the sensitivity has been lower (62.5% – 84%) [1-3].

The most significant consequence of premature discontinuation of cervical spine immobilization is neurological injury. Prolonged immobilization, however, is associated with morbidity as well. Decubitus ulcers, increased cerebrospinal fluid pressure, pain and pulmonary complications have all been described with prolonged immobilization of the cervical spine [4-6]. The single most common cause of missed cervical spine injury appears to be failure to adequately visualize the region of injury. This can be caused by failure to obtain radiographs, or by making judgments on technically suboptimal films. This occurs most commonly at the extremes of the cervical spine, the occiput to C2 and at the C7-T1 levels [7-9]. Visualising the C7-T1 junction is therefore extremely important. In order to improve the visualization of this region, various additional imaging modalities have been recommended with the Swimmer's view being the commonest [10-13].

There has been no study in the literature that assesses the adequacy of the Swimmer's view on its own. Our study aims to do this. There are studies comparing the supine oblique views and the Swimmer's view but the results are varied [14,15].

Our study showed that 45% of the Swimmer's view radiographs were inadequate. Although this study has its limitations (retrospective study, small sample), in light of our findings we strongly believe that the Swimmer's view should not be used as the imaging modality of choice to visualize the C7-T1 junction prior to clearing the cervical spine for removal of immobilization. In order to increase the sensitivity of the radiographic assessment of the cervical spine in trauma patients, we recommend a CT or MR evaluation of the cervical spine. The utility of these imaging modalities for this purpose is well documented in the literature [10-13]. If there is a high level of clinical suspicion the sensitivity and specificity of a CT or an MRI scan will be increased. The efficacy of a multislice CT or an MR for screening of the cervical spine in obtunded patients is well documented [16,17]. These modalities have been found to be superior to dynamic radiography and plain radiography [18,19]. MR imaging detects ligamentous injuries in the cervical spine which can be missed on CT scans [19,20].

In light of these facts and the findings from our study, should we be performing the Swimmer's view at all? Is it better to perform a CT evaluation of the cervical spine prior to clearing the cervical spine?

## Conclusion

The Swimmer's view is generally considered as the commonest additional view to supplement an inadequate lateral cervical spine radiograph to visualize the cervical spine [15]. Adequate visualization of the entire cervical spine is essential in a trauma patient to prevent neurological injury due to hasty removal of immobilization in a missed cervical spine injury. We found the Swimmer's view to be unreliable for this purpose and recommend using other imaging modalities like CT or MR scans.

## List of abbreviations

CT – Computed Tomography

MR – Magnetic Resonance

## Competing interests

The author(s) declare that they have no competing interests.

## Authors' contributions

UR, the main author was responsible for conducting the study, acquisition, analysis and interpretation of the data and preparing the manuscript.

RSUY, the co-author was responsible for literature review, data acquisition and has approved the final draft.

SSB, the senior author was responsible for supervising the study, proof reading of the manuscript and has approved the final draft of the manuscript.

## Pre-publication history

The pre-publication history for this paper can be accessed here:


